# Risk Factors and Prediction of Leptospiral Seropositivity Among Dogs and Dog Handlers in Malaysia

**DOI:** 10.3390/ijerph16091499

**Published:** 2019-04-28

**Authors:** Soon Heng Goh, Rosnah Ismail, Seng Fong Lau, Puteri Azaziah Megat Abdul Rani, Taznim Begam Mohd Mohidin, Faiz Daud, Abdul Rani Bahaman, Siti Khairani-Bejo, Rozanaliza Radzi, Kuan Hua Khor

**Affiliations:** 1 Department of Veterinary Clinical Studies, Faculty of Veterinary Medicine, Universiti Putra Malaysia, Serdang 43400, Malaysia; chrisvangoh_14@hotmail.com (S.H.G.); lausengfong@upm.edu.my (S.F.L.); rozanaliza@upm.edu.my (R.R.); 2 Occupational Health Unit, Community Health Department, Faculty of Medicine, Universiti Kebangsaan Malaysia, Bangi 43600 UKM, Malaysia; drrose@ppukm.ukm.edu.my (R.I.); drfaizdaud@gmail.com (F.D.); 3 Department of Companion Animal Medicine and Surgery, Faculty of Veterinary Medicine, Universiti Putra Malaysia, Serdang 43400, Malaysia; azaziah@upm.edu.my; 4 Institute of Biological Sciences, Faculty of Science, University of Malaya, Kuala Lumpur 50603, Malaysia; taznim@um.edu.my; 5 Department of Pathology and Microbiology, Faculty of Veterinary Medicine, Universiti Putra Malaysia, Serdang 43400, Malaysia; ranibahaman3@gmail.com (A.R.B.); skhairani@upm.edu.my (S.K.-B.)

**Keywords:** seroprevalence, MAT, leptospirosis, dog handlers, working dogs, shelter dog

## Abstract

This study determined the potential risk factors that may contribute to seropositivity among dogs and dog handlers from working dog and dog shelter institutions. Data was collected from dogs (*n* = 266) and dog handlers (*n* = 161) using a standardised guided questionnaire. Serum obtained from the dogs and dog handlers was tested using the microscopic agglutination test (MAT). A logistic regression analysis was used to predict leptospiral seropositivity of dogs and dog handlers based on potential risk factors. A total of 22.2% of dogs and 21.7% of dog handlers were seropositive. The significant predictors for the dogs’ seropositivity were presence of rats (OR = 4.61 (95% CI: 1.05, 20.33), *p* = 0.043) and shared common area (OR = 5.12 (95% CI: 1.94, 13.46), *p* = 0.001) within the organisation. Significant predictor for dog handler seropositivity was contact time with the dogs of more than six hours/day (OR = 3.28 (95% CI: 1.28, 8.40), *p* = 0.013) after controlling for the effect of other risk factors such as small mammal contact, rat infestation at home, flooding at housing area (within three months) and urban locality. The exposure to various disease sources identified poses risk to dogs and dog handlers. Risk could be reduced with adequate application of protection at work while handling dogs and thus limiting contact with these sources and reducing exposure to infection.

## 1. Introduction

Leptospirosis, is a neglected re-emerging global zoonosis common in the warm and humid tropics and subtropics [1]. Reported cases span from South America and the Caribbean to Southeast Asia and Oceania [2]. Globally, 58,900 people were estimated to succumb annually from 1.03 million reported cases [3]. In Malaysia, an upward trend has been observed, from 263 cases reported in 2004 to 5370 cases in 2015 with a sudden spike in 2014 with 7806 cases [4], which resulted from heavy rainfall and massive flooding during the rainy season. However, the annual human mortality rate fluctuated [5]. 

Leptospirosis affect mammals, such as livestock animals (pigs, cattle, horses, goats and sheep), companion animals (dogs), small mammals (rats, mice and raccoons) and humans [6]. Rats remained the major natural reservoir responsible for disease maintenance of the spirochaetes by contaminating the environment via urine secretion [7,8]. Human outbreaks often occur from direct or indirect contact with leptospires contaminated environments (soil and water), e.g., after a massive flood and post-recreational activities [9]. Leptospirosis has been highlighted as an occupational disease among workers in the livestock and agriculture industry, as well as sewage management workers [10,11,12]. There is high likelihood that these groups of workers were exposed to unhygienic environments with possible direct or indirect contact with reservoir animals (i.e., rats, dogs and livestock) and urine-contaminated soil and water [13]. 

In Kenya, seropositive cases among kennel workers reported were likely due to close contact with infected or carrier dogs [14]. Little is known about the true potential of dogs as carriers but a wide range of seropositivity levels among dogs in different countries were reported between 5.5% to 71.1%, with various serovars detected [15,16]. From current knowledge, there is still limited investigation looking into the probable risk factors threatening dogs and dog handlers. Therefore, the potential risk factors that may have contributed to seropositivity among dogs and dog handlers from working dog and dog shelter organisations was determined in this study. Findings obtained would improve the preventive measures implemented, allowing a better working environment for dogs and their handlers, thus preventing future occurrences. 

## 2. Materials and Methods 

### 2.1. Study Design 

This cross-sectional study was carried out over a five month period among working dog and dog shelter organisations. Eight organisations consisting of four working dog and four dog shelter organisations from two states (Johore and Selangor) in Malaysia were visited. At each location, both dogs and their handlers were conveniently recruited. Informed consent was obtained from each participant prior to questionnaire distribution, anonymity was assured and all the information obtained was treated with confidentiality for the purpose of research. 

### 2.2. Blood Collection

Approximately 3 mL of blood samples were collected from the dogs’ cephalic veins, whereas 5 mL of blood was withdrawn from the dog handlers’ brachial vein. Blood samples were collected in plain vacutainer tubes, stored in a chiller box (2 to 4 °C) and immediately transported to the Bacteriology Laboratory of Faculty of Veterinary Medicine, Universiti Putra Malaysia. Serum was harvested and archived at −20 ℃ for further serological analysis.

### 2.3. Microscopic Agglutination Test (MAT)

Serological testing using MAT was carried out according to the established methods [17]. All the serum samples were tested against 20 leptospiral serovar antigens to quantify the level of agglutinating antibodies in the body. The selection of leptospiral antigens were based on the commonly found pathogenic (either found in dogs or humans or shared by both) and a saprophytic serovar. The serovars chosen were Icterohaemorrhagiae, Canicola, Pomona, Bataviae, Australis, Tarassovi, Autumnalis, Pyrogenes, Hebdomadis, Hardjo, Lai, Copenhageni, Celledoni, Grippotyphosa, Cynopteri, Ballum, Hardjobovis, Javanica, Malaysia and Patoc. All antigens were obtained from Leptospirosis Reference Laboratory, Queensland Health, Queensland, Australia. The cut-off titre of 1:50 was used for the dog handlers to determine exposure or post-infection towards leptospirosis, while for the dogs, 1:100 was used based on the standard recommendation set by the OIE [17]. The sample was considered seropositive if there were <50% free leptospires and >50% agglutination when compared to the positive control (hyperimmune serum) and negative control (antigen only).

### 2.4. Questionnaires

A validated self-administered questionnaire was developed. All the questionnaire’s items were consensually agreed by eight veterinary experts using Fuzzy Delphi technique, which utilised two concepts known as Triangular Fuzzy Numbers and Defuzzification Process. The items in this questionnaire were accepted based on the Fuzzy Delphi Threshold value ≤ 0.2. The questionnaire was made available in a bilingual (Malay and English) format. 

The questionnaire collected information of the dog handlers and their dogs. From the dogs, (i) demographics (age, gender, breed, health status and vaccination records) and (ii) environment factors (i.e., small mammal contact, rat contact and shared common area). 

From the dog handlers, (i) sociodemographic details (age, gender, race, health status and job scope), (ii) occupational factors (i.e., possible contact with small mammal such as squirrels, civets etc.; possible contact with rats in the environment; daily contact time with dogs), and (iii) non-occupational factors (i.e. recreational activity, small mammals exposure at home, rat breeding area at home, locality of monsoon drain and occurrences of flash floods) were obtained. 

All the responses to the risk factors were dichotomously recorded. Additional information such as kennel cleanliness, feeding regime, food storage area and surrounding of the area was compared between both dog shelter and working dog organisations. 

### 2.5. Definition of Risk Factors

The dog risk factors used for analysis for the dogs were as follows; contact with small mammals, contact with rats, shared common area, kennel cleanliness and location of organisation. Possible dog contact with small mammals and rats was considered positive if there was exposure during and off work. If there was presence of shared common area in the organisation’s premises for the dogs, it was considered positive. Responses for the cleanliness of the kennel cleanliness (clean/dirty) were noted during the site visit and “dirty” was considered as a positive response. Organisational locality (either urban/rural) was noted where urban was considered as a positive response. 

The dog handlers’ risk factors were further sub-grouped as occupational (work-related) and non-occupational (non-work related). Occupational factors included in this study were contact with small mammals, contact with rats, daily contact time with dog and location of the organisation. Contact with small mammals and rats were referred to as contact or exposure during working hours at the organisation and offsite locations during operations and was considered positive if there was contact. It was also understood that if their dogs were exposed, their handlers would be too. The daily contact time with dogs was determined based on the duration of time (either <6 or >6 hours/day) the dog handlers spent with the dogs under their care for the past 12 months, and response >6 hours/day was considered positive. Location of organisation (urban/rural) was noted, where urban was considered as a positive response.

Non-occupational factors were activities that dog handlers had beyond working hours and unrelated to his/her occupational responsibilities. The risk factors evaluated were recreational activity involvement, presence of small mammals in the housing area, presence of rat breeding ground near the house, occurrences of flooding within the last three months and any monsoon drain situated within 15 m radius from their house. Recent recreational activity involvement was considered present if they responded to the item. The presence/exposure to small mammals and rat breeding (infestation) at their housing area was considered positive if there was exposure. The experience of flooding at the housing area (past three months) and presence of monsoon drains 15 m away from their house, it was considered positive if the dog handlers responded “Yes.”

### 2.6. Statistical Analysis

Data was tabulated and analysed using IBM SPSS version 23 (IBM, Armonk, NY, USA). All the information on the dogs, dog handlers, the risk factors and seroconversion status were descriptively analysed. The Cronbach’s alpha of the measured items was more than 0.8 which indicated a good internal consistency reliability. Logistic regression was applied to develop the prediction model of seropositivity for dogs and dog handlers. Seropositivity was dummy coded as 1. All factors possessing *p*-values of <0.25 after simple logistic regression were submitted for multiple logistic regression models using a backward likelihood ratio (LR) elimination method to obtain the final model. The final model was chosen based on the largest Nagelkerker r^2^ and majority of statistical model assumptions were met. The goodness of statistical model assumption was met if (i) Hosmer-Lemeshow test was not significant (*p* > 0.05); (ii) overall percentage from classification table > 70% and (iii) area under the Receiver Operating Characteristic curve > 0.70. Interaction and multi-collinearity were evaluated before accepting the final model. Statistical significance was determined by a *p*-value ≤ 0.05 due to small sample size.

### 2.7. Ethical Approval

The study obtained two ethical clearance approval. Institutional Animal Care and Use Committee (UPM/IACUC/AUP-R091/2016) allowed recruitment and handling of the dogs, whereas Research Ethics Committee (UKMPPI/111/8/JEP-2016-494) allowed recruitment, blood collection and completion of questionnaire from the dog handlers Consent from each organisation and every dog handler was obtained prior to the start of the study. Information obtained was confidential and to be only used for research purposes.

## 3. Results

In this study, 266 overtly healthy and vaccinated dogs (females, *n* = 79; males, *n* = 187) with mean age of 3 (age range: 1- to 11-years-old) were recruited. Majority of the dogs (72.6%, *n* = 193) were from the dog shelters, consist of local mixed-breed (*n* = 191/193), except for a Labrador and a German Shepherd Dog (GSD). The working dog organisations had various breeds, which consist of GSD (*n* = 18/73), Malinois (*n* = 10/73), English Springer Spaniel (*n* = 2/73), Cocker Spaniel (*n* = 9/73) and Labrador Retriever (*n* = 36/73). Based on obtainable records, only 152 dogs (working, *n* = 103; shelter, *n* = 49) had an up-to-date annual vaccination (<1 year). Of those 152 dogs, 113 dogs received a bivalent vaccine (Icterohaemorrhagiae and Canicola), while the remaining 39 dogs received quadrivalent vaccine (Icterohaemorrhagiae, Canicola, Pomona and Grippotyphosa). 

A total of 161 dog handlers (males, *n* = 152; females, *n* = 9) with an average age of 30 (age range: 20- to 61-years-old) claimed that they were healthy upon blood sampling. Majority of the dog handlers’ ethnicities were of Bornean indigenous (*n* = 56), followed by Indian (*n* = 54), Chinese (*n* = 12) and Malay (*n* = 13). The remaining were foreigners (*n* = 26), consisting of Indonesian (*n* = 20), Myanmarese (*n* = 5) and Pakistani (*n* = 1). Job scopes varied between both groups. From the working dog organisations, 75 out of 128 dog handlers had daily duties of dog handling (58.6%), 41.4% (*n* = 53/128) had administrative duties and 0.9% (*n* = 2/128) were kennel men. At times, the dog handlers carried out the tasks of kennel men as needed. A different scenario was observed at the dog shelters. The kennel men were involved directly with dog handling and managing the kennels whereas administrative staff were limited to their management roles. Of the 33 shelter dog handlers, 15.2% (*n* = 5) had administrative duties and 84.8% (*n* = 28) were kennel men. Only in some shelters, administrative staff may assist/or undertake the kennel men duties during times of high workload. The frequency of exposure of dogs and dog handlers to risk factors was as shown in Table 1.

**Figure 1:**
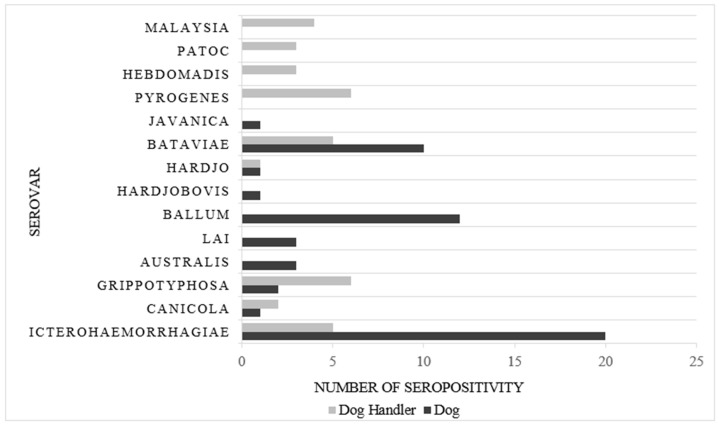
Distribution of leptospiral serovars among dogs (*n* = 266) and dog handlers (*n* = 161).

The leptospiral seropositivity among dogs was 22.2% (*n* = 59/266) (titres ranged: 1:100–1:800). Ten leptospira serovars comprising of Icterohaemorrhagiae, Canicola, Bataviae, Australis, Hardjo, Lai, Grippotyphosa, Ballum, Hardjobovis and Javanica were detected among the dogs (refer to Figure 1). A similar seropositivity percentage was detected among dog handlers (21.7%; *n* = 35/161; titres ranged: 1:50–1:200). Nine leptospira serovars comprising of Icterohaemorrhagiae, Canicola, Bataviae, Hardjo, Grippotyphosa, Pyrogenes, Hebdomadis, Patoc and Malaysia were detected among the dog handlers (refer to Figure 1).

A logistic regression was performed to ascertain the effects of rat contact and shared common area on the likelihood that dogs were seropositive with small mammal contact, kennel cleanliness and urban locality as controlled variables. The model was statistically significant χ^2^ (2) = 18.69, *p* < 0.001. The model explained 10.4% of the variance in the leptospiral seropositivity and correctly classified 77.8% of cases. Shared common area was 5.12 times more likely to contribute towards seropositivity than isolated area. Dogs with a history of rat contact was 4.61 times more likely to be seropositive than those without (refer to Table 2).

A similar analysis was conducted for the dog handlers to ascertain the effects of contact time with dog of >6 hours/day on the likelihood that dog handlers were leptospiral seropositive with urban locality and non-occupational exposure, i.e. small mammal contact, rat infestation surrounding home and flash flooding at their housing area in the last three months as controlled variables. The model was statistically significant χ^2^ (5) = 34.77, *p* < 0.001. The model explained 29.9% of the variance in the leptospiral seropositivity and correctly classified 86.3% of cases. Dog handlers with contact time of >6 hours/day working with dogs were 3.28 times more likely to show seropositivity than those with contact time of <6 hours/day (refer to Table 3). 

## 4. Discussion

Human leptospirosis has always been associated with exposure to contaminated environment exposure i.e., from flooding and outdoor recreational activities [18,19]. Recently, associations to certain occupations involving animal contact, sewage management and agricultural work (farm and plantation) were reported [10]. These workers often worked in dirty environments containing soil and water likely contaminated with *Leptospira* spp., and with possible reservoir animals (rats, dogs and livestock animals) contact [13]. Therefore, the direct or indirect animal-human interaction may contribute to disease transmission and poses a zoonotic risk. Dogs can be subclinically infected in which they may appear healthy, and may have contributed to increased risk of infection due to close contact with dog handlers [20]. 

From current knowledge, investigation looking into the probable risk factors threatening dogs and dog handlers has not been investigated. Predicting leptospiral seropositivity through potential risk factors identification would be useful in disease management. Seroconversion may not indicate disease as it can be due to vaccination or post-exposure. From the ten *Leptospira* spp. serovars detected among dogs, the majority were Icterohaemorrhagie, Ballum and Bataviae. The detection of serovar Icterohaemorrhagiae could be due to post-vaccination but alarmingly, the detection of non-vaccinal serovars Ballum and Bataviae could suggest direct/indirect transmission from animal reservoirs to dogs. Disease may occur despite vaccination because it produces only serovar-specific immunity [21]. Infected dogs could be potential carriers if untreated or treated unsuccessfully. The actual disease status of the dogs in this study were not investigated due to limitations in obtaining urine samples.

Rats have been vastly reported as the main reservoir harbouring a wide range of *Leptospira* spp. that causes leptospirosis in both animals and humans [22]. Contaminated environments increased the risk dogs’ and their handlers’ contact/exposure with rats hence, contributing to a high probability of seropositivity. Detection of high seropositivity (22.2%, *n* = 59/266) among dogs could be due to exposure to harsh environments and is in disagreement with past reports involving indoor dogs with minimal risk of exposure [23,24]. The increased risk affirmed the significant role of rats in the transmission through possible direct or indirect contact leading to high serodetection among dogs. The maintenance role of rats may have enabled environmental persistence [25] but was not further investigated.

All the organisations had a multipurpose shared common area within the premise. At the working dog organisations, it was used for daily training activities (i.e., search and rescue, tracking and apprehension skills of the dogs). The training areas were fenced-up open field or a covered area with large training obstacles. At the dog shelters, different group of dogs were rotated within multipurpose shared common area for exercise and walks during activities of kennel cleaning and feeding. It was observed that these areas were located near natural water sources, such as waterfalls, rivers or ponds with possible access. Therefore, if a dog in the group was infected, that dog would become the potential infection source contaminating these areas, therefore exacerbating the possibility of disease dissemination. 

Human overcrowding affecting hygiene and sanitation was a risk factor for human leptospirosis [26]. Overcrowding of dogs was evident at the shelters, unlike working dogs organisations, the dogs were individually housed. In this study, shelter dogs were housed in large numbers due to limited space, thus increasing possible dog-to-dog contact [27], which may increase risk of disease transmission. Besides that, overpopulated dog shelters managing high manure output with lack of manpower may lead to the poor kennel sanitation and possibly have a similar potential impact on the dogs [12,25]. 

Four (Grippotyphosa, Icterohaemorrhagiae, Pyrogenes and Bataviae) main serovars detected among dog handlers were commonly associated with the rats or small mammals [6,28]. In this study, rat or small mammal contact was not a significant predicting risk factor towards high seropositivy among dog handlers, thus suggesting other possible sources, such as dogs. Dogs have been reported to be reservoirs mainly for *Leptospira interrogans* serovar Canicola [6]. Further analysis found that prolonged dog handler-dog contact time increases the risk of seropositivity, suggesting a major role of dogs. The unknowing handling of infected asymptomatic dogs [29] puts the dog handlers at risk from the leptospires shedding [30,31]. In this study, both dogs and dog handlers were seropositive towards five serovars (Grippotyphosa, Icterohaemorrhagiae, Canicola, Hardjo and Bataviae). Alarmingly, two (Hardjo and Bataviae) serovars were not available in the canine vaccination protocol. This finding may suggest that the dogs may be at risk of infection and may indirectly increase the risk of infection for the dog handlers. However, possibilities of direct infection of dog handlers during work in harsh environments could only be speculated.

In terms of the location of the organisation, dogs from organisations in urban locality had an increased risk of leptospiral seropositivity compared to rural [32,33,34]. This could be attributed to the coexistence of reservoirs animals and dogs [9,18]. Rats were claimed observed present within the organisation’s compound possibly scavenging for food from dog feeding areas (as food provided ad libitum) and unmanaged garbage disposal areas. However, the seropositive dog handlers from urban organisations (one dog shelter and three working dog organisations) were not as many as the dogs from the same location, as they were observed to implement good hygiene and sanitation practices. In fact, more dog handlers from the rural locality were seropositive from being situated on agricultural land (i.e., oil palm plantations, jungle and forest regions), which may have increased contact/exposure to animal reservoirs. It was also speculated that working dogs were possibly exposed to *Leptospira* spp. during operational work/task assigned (i.e. search and rescue, bomb detection, arson, cadaver retrieval etc.) in uncontrolled harsh environments (i.e. natural disaster sites, jungle regions, areas with large water bodies and urban areas) [11]. These working dogs are part of the government authorities with special task for enforcement, protection, search and rescue.

Dog handlers may have been exposed to contaminated non-work-related environments. Based on the information obtained, seroconversion due to rat infestation at the dog handlers’ housing area as a probable source of exposure could not be ruled out. The presence of a monsoon drain within 15 m radius from their homes as rat breeding ground may pose a threat, similar to scenarios in India [23]. Trapping of rats at these areas would elucidate this observation further but was not possible at the time of the study. High humidity in tropical countries like Malaysia resulting from year-round rainfall cultivates *Leptospira* spp. growth which becomes prominent during rainy seasons with increased rainfall [34,35]. Recent flood experience especially in areas with poor drainage and irrigation systems may further increase the chance of seroconversion [36] due to the scattering of organisms over a wider geographical region allowing persistence in water and soil [37,38,39]. 

## 5. Conclusions

In conclusion, history of rat contact/exposure and shared common area within the organisation may contributed to seropositivity among dogs, while prolonged contact time of >6 hours/day with dogs was the significant contributed risk factors for the dog handlers. This study affirmed that the exposure to disease source (animal or environment) playing a paramount role in the dissemination of leptospirosis among dogs and dog handlers. The unique setting of these organisations could become a challenge in efforts to curb leptospirosis. Breaking the chain of infection with the usage of adequate personal protective equipment (PPE) upon dog handlings at the organisation level would reduce the risk of leptospiral infection. It requires targeted control and preventive measures at the organisation and awareness of the risk among dog handlers in order to prevent potential future occurrences.

## Figures and Tables

**Table 1:** Descriptive analysis of dogs’ and dog handlers’ exposure to risk factors identified in this study.

Risk Factors	Frequency	%
Dogs’ factor (*n* = 266)		
Had small mammal contact	219	82.3
Had rat contact	242	91.0
Shared common area	200	75.2
Dirty kennel	227	85.3
Urban location	126	47.4
Dog handlers’ factor (*n* = 161)		
Occupational:		
Had small mammal contact	57	35.4
Had rat contact	45	28.0
Contact time with dog > 6 hours/day	80	49.7
Urban location	111	68.9
Non-occupational:		
Had recreational activity in last 3 months	69	42.9
Rat infestation at home	85	52.5
Small mammal around the housing area	105	65.2
Had flash flood at the housing area in last 3 months	15	9.3
Monsoon drain within 15m radius from the house	63	39.1
MAT Seropositivity		
Dogs (*n* = 266)	59	22.2
Working Dog (*n* = 73)	17	6.4
Shelter Dog (*n* = 193)	42	15.8
Dogs handlers (*n* = 161)	35	21.7
Working Dog (*n* = 128)	15	9.3
Shelter Dog (*n* = 33)	20	12.4

**Table 2:** Risk factor affecting dogs’ leptospiral seropositivity from working dog and shelter dog organisations.

Variables	Simple Logistic Regression			Multiple Logistic Regression ^a^		
	b	Crude OR (95% CI)	*p*-Value	b	Adjusted OR (95% CI)	*p*-Value
Had small mammal contact	−0.42	0.66 (0.29, 1.52)	0.331			
Had rat contact	1.22	3.39 (0.77, 14.85)	0.105	1.53	4.61 (1.05, 20.33)	0.043
Share common area	1.51	4.51 (1.72, 11.83)	0.002	1.63	5.12 (1.94, 13.46)	0.001
Kennel cleanliness	0.23	1.25 (0.57, 2.75)	0.574			
Urban location	0.80	2.23 (1.23, 4.04)	0.008			

OR = Odd Ratio, CI = Confidence Interval; ^a^ Backward Likelihood Ratio (LR) Multivariate Multiple Logistic Regression was applied. Multicollinearity and interaction were checked. Hosmer-Lemeshow test (*p* > 0.05), classification table (overall correctly classified percentage = 77.8%) and area under the ROC curve (0.78) were applied to check the model fitness, r^2^ = 0.104.

**Table 3:** Occupational and non-occupational risk factors affecting dog handlers’ leptospiral seropositivity from working dog and shelter dog organisations.

Risk Factors		Simple Logistic Regression			Multiple Logistic Regression ^a^		
		b	Crude OR (95% CI)	*p*-Value	B	Adjusted OR (95% CI)	*p*-Value
**Occupational**	Small mammal contact	1.48	4.40 (1.99, 9.68)	<0.001	0.79	2.21 (0.91, 5.40)	0.082
	Had rat contact	1.85	6.38 (2.84, 14.32)	<0.001	-	-	-
	Contact time with dog > 6 hours daily	1.53	4.65 (1.96, 11.04)	<0.001	1.19	3.28 (1.28, 8.40)	0.013
	Urban area	−1.14	0.32 (0.15, 0.70)	0.004	−0.98	0.38 (0.16, 0.91)	0.029
**Non-occupational**	Had recreational activity	−0.46	0.63 (0.29, 1.38)	0.249	-	-	-
	Rat infestation surrounding home	1.01	2.75 (1.22, 6.20)	0.015	0.91	2.44 (0.95, 6.52)	0.065
	Small mammals around the house	0.70	2.01 (0.84, 4.78)	0.115	-	-	-
	Had flash flood at home in last 3 months	1.45	4.25 (1.38, 3.10)	0.012	1.40	4.04 (1.08, 15.16)	0.038
	Had monsoon drain 15m from house	−0.27	0.77 (0.35, 1.68)	0.507	-	-	-

OR = Odd Ratio, CI = Confidence Interval; ^a^ Backward Likelihood Ratio (LR) Multiple Logistic Regression was applied. Multicollinearity and interaction was checked. Hosmer-Lemeshow test (*p* > 0.05), classification table (overall correctly classified percentage = 86.3%) and area under the ROC curve (0.78) were applied to check the model fitness, r^2^ = 0.299.
